# The volatile and heterogeneous gut microbiota shifts of COVID‐19 patients over the course of a probiotics‐assisted therapy

**DOI:** 10.1002/ctm2.643

**Published:** 2021-12-28

**Authors:** Chunyan Wu, Qian Xu, Zhan Cao, Dengdeng Pan, Ying Zhu, Sheng Wang, Danping Liu, Zhigang Song, Wei Jiang, Yumeng Ruan, Yongkun Huang, Nan Qin, Hongzhou Lu, Huanlong Qin

**Affiliations:** ^1^ Institute of Intestinal Diseases Shanghai Tenth People's Hospital Tongji University School of Medicine Shanghai China; ^2^ Department of Critical Care Medicine Shanghai Tenth People's Hospital Tongji University Shanghai China; ^3^ Department of Infectious Disease Shanghai Public Health Clinical Center Shanghai China; ^4^ Realbio Genomics Institute Shanghai China; ^5^ Department of Pediatrics The First Affiliated Hospital of Kunming Medical University Kunming China; ^6^ Yunnan Key Laboratory of Clinical Medicine Kunming China

Dear Editor,

It has been reported that up to half but as low as 3% of COVID‐19 patients had gastrointestinal symptoms (e.g., diarrhoea, nausea, and vomiting)^1^, ^2^, ^3^ and substantial gut microbiota shifts,^4^, ^5^ a testament to the importance of the microbial community in the therapeutic responses and prognosis of the disease. Nevertheless, given the seriously rapid progression of COVID‐19,^6^ it is important to investigate the microbial dynamics during the illness and convalescence stages. Here, we employed a probiotics‐assisted therapy to treat a group of COVID‐19 patients, and their dynamics of gut microbiota and clinical manifestations were monitored throughout the treatment.

We recruited 13 COVID‐19 patients, 15 healthy controls (HCs), and 15 non‐COVID‐19 pneumonia controls (NC‐PCs) (Table S1). Decreases of inflammatory indicators were common in the COVID‐19 patients after the treatment (determined by > two‐fold change, Table S2). Analysis of 16S rRNA gene sequencing revealed that in comparison with the HCs and NC‐PCs, the COVID‐19 patients exhibited a different microbial structure (ANOSIM, *p* < .05, Figure 1A) and a reduced microbial diversity (Shannon index, *p* < .05, Table S3) and that dominance with one or two genera was apparent in individual COVID‐19 patients (Figure 1B, Figure S1). Compared with the HCs, the COVID‐19 patients exhibited reduced relative abundances of Firmicutes at the phylum level and 21 genera (FDR < .05; Table S4). Differences in transcriptional activities of gut microbiota were also observed, as the COVID‐19 patients were characterized by the augmented presence of 322 species, including *Escherichia coli*, *Salmonella enterica*, *Staphylococcus auricularis*, *Klebsiella pneumoniae* and *Enterococcus faecium*, and decreased presence of 279 species, including *Bacteroides vulgatus*, *Faecalibacterium prausnitzii* and *Eubacterium eligens* (FDR < .05; Figure 1C and Table S5). There was some noticeable coincidence between the taxonomic and transcriptional shifts, as evidenced by the genera or their affiliating species, such as *Faecalibacterium*, *Enterococcus* and *Rhodococcus*. Examination of strain‐level variations revealed considerable below‐species heterogeneity of *Escherichia coli* in the COVID‐19 patients (Figure S2). Overall, our analyses revealed that the COVID‐19‐associated gut microbiome perturbations exhibited great heterogeneity among individual patients.

**FIGURE 1:**
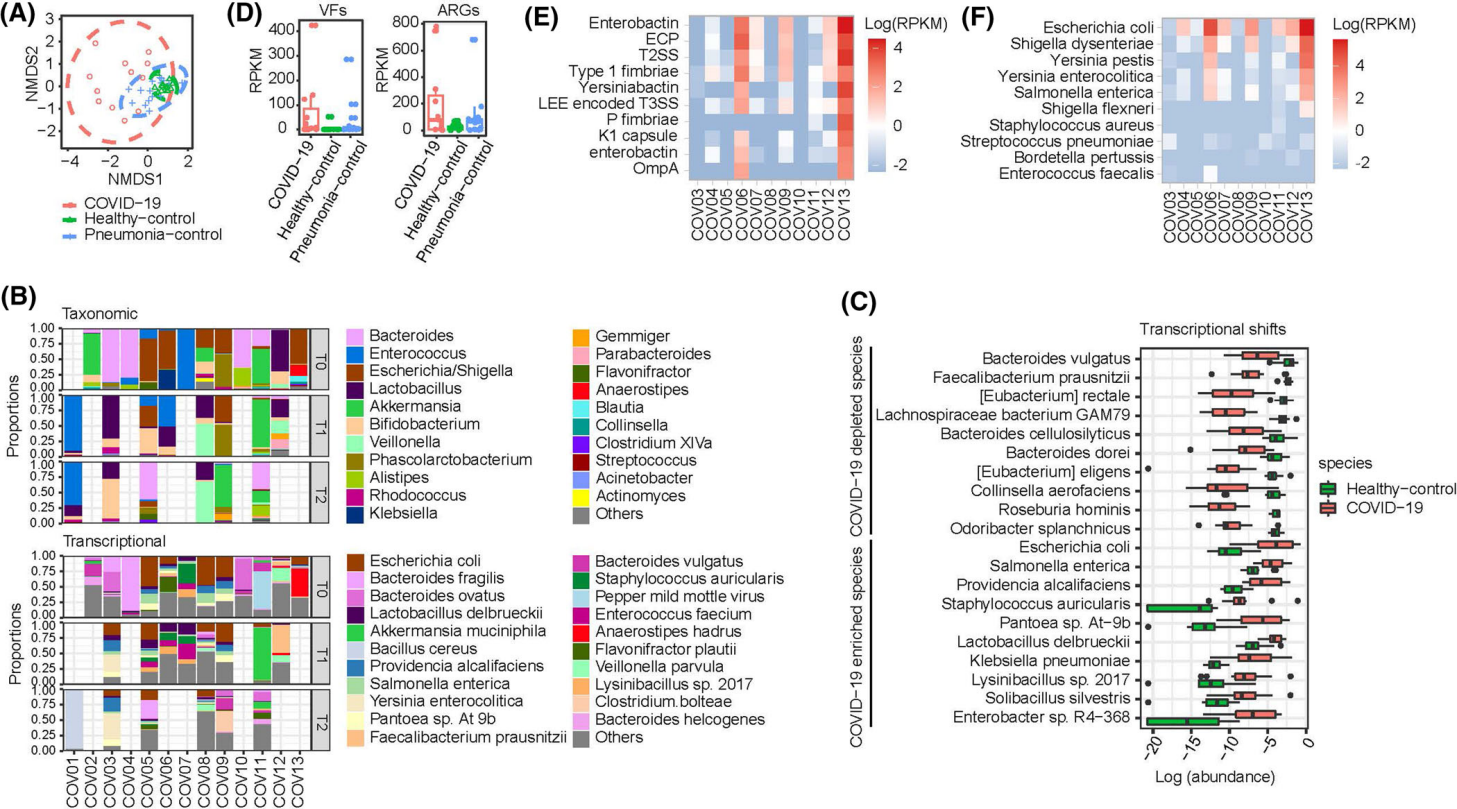
Gut microbiome alterations in patients with COVID‐19 and longitudinal changes over the course of therapy. (A) Microbial community alterations in COVID‐19, generated by NMDS (Non‐metric multidimensional scaling) plot based upon Bray‐Curtis dissimilarities. The microbial compositions were compared between healthy controls (*n* = 15), COVID‐19 patients (*n* = 12), and pneumonia controls (*n* = 15). (B) Relative abundance of top 20 genera and transcriptionally active species in COVID‐19. (C) Top 20 transcriptionally active species that were associated with COVID‐19 (FDR < 0.05). (D) Box plot showing the RPKM values of virulence factors (VFs) and antibiotic resistance genes (ARGs). (E,F) Box plots showing the top 10 most abundant virulence factors (E) and contributing species of virulence factor (F)

To understand the microbial functional characteristics, we analyzed the expression profiles of pathways, virulence factors and antibiotic resistance genes. The COVID‐19 patients were characterized by enrichment of 11 MetaCyc pathways including beta‐Lactam resistance, Biofilm formation‐*Escherichia coli*, and Bacterial invasion of epithelial cells as well as depletion of 86 MetaCyc pathways, most of which related to the metabolism of amino acids, lipids, and carbohydrates (Table S6). In addition, the expression levels of virulence factors and antibiotic resistance genes (ARGs) in the COVID‐19 patients were significantly higher than both the HCs and NC‐PCs (Figure 1D–E). Lastly, the top five transcriptionally active virulence factors (Figure 1E; Table S7), top five contributing species of virulence factors (Figure 1F; Table S8), and top 10 most abundant antibiotic resistance targets (Figure S3A; Table S9) and ARGs (Figure S3B; Table S10) collectively indicated the prominent presence of virulence factors and their contributing pathogens/pathobionts in the COVID‐19 patients.

To assess the clinical relevance of the observed gut microbiota disturbances, spearman's ranks were computed to reveal the correlations of clinical indicators with upper airway or gut microbiota features (Figure 2A). In the upper airway, *Bacteroides*, *Akkermansia* and *Enterococcus* showed negative associations with CD3 and CD4. In the gut, *Rhodococcus* and *Acinetobacter* showed negative associations with CD3, CD4, CD45, haemoglobin (Hb) concentrations, and red blood cells (RBC); *Bacteroides* and *Veillonella* displayed positive association with haemoglobins, RBC, and CD3, respectively, as did *Enterococcus* with plasma concentration of carbon dioxide. The expression levels of bacteria virulence factors (e.g., Yersiniabactin, Salmochelin, Shu, and SgrA) were negatively correlated with CD8 (Figure 2B), whereas multiple antibiotic resistance genes were negatively associated with CD3 (Figure 2C). These findings indicated that gut and airway microbiota shifts were linked to the clinical manifestations of inflammation, possibly related to COVID‐19.

**FIGURE 2:**
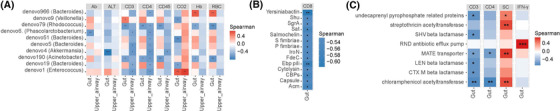
Correlation of features of gut microbiota with clinical indicators. (A–C) Pairwise Spearman's correlation matrix of the OTUs (A), antibiotic resistance genes (B), and virulence factors (C) associated with different clinical indicators (*adjusted *p* < .05; **adjusted *p* < .01)

To investigate the dynamics of the gut microbiome in the COVID‐19 patients during the treatment, we compared the taxonomic data among the baseline (T0, first sampling date before the probiotics‐assisted therapy), 7‐day posttreatment (T1), and 14‐day post‐treatment (T2) for each patient. Of the 12 recruited COVID‐19 patients, stool samples both before and after the therapy could only be collected from eight individuals (Figure 1B). At the end of the treatment, six out of the eight COVID‐19 patients (75%) showed a partial microbial compositional “recovery”, evidenced by a decrease of Bray‐Curtis (BC) dissimilarity to the healthy group (Figure 3A). This alteration was accompanied by substantial taxonomic shift, including a reduction of COVID‐19‐enriched genera (e.g., *Rhodococcus* in 71% patients) and an increase of COVID‐19‐depleted genera (e.g., *Clostridium* XlVa in 57% patients), respectively (> two‐fold change, Table S11), for which some probiotic species (e.g., *Lactobacillus paracasei* and *Lactobacillus plantarum*) showed prominent influences (Figure S4, Adonis test, FDR < .05). This partial “normalization” was paralleled by a similar trend in the microbial transcriptome. After treatment, 58/322 of the species featuring COVID‐19‐associated transcriptional elevation exhibited at least two‐fold reduction in more than 75% of the COVID‐19 patients (e.g., opportunistic pathogen *Escherichia coli* (88%) and *Klebsiella pneumoniae* (75%)), whereas 22/279 of the species that had COVID‐19‐associated transcriptional reduction exhibited at least two‐fold increase in more than 65% of the COVID‐19 patients (e.g., the major gut commensal bacterium *Faecalibacterium prausnitzii* (65%) (Table S12, Figure 3B). An important finding of the longitudinal data was that there were great inter‐timepoint compositional variations of the gut microbiome in most COVID‐19 patients (Figure 1B). For example, the top taxa of patient COV05, COV06, and COV08 was *Escherichia/Shigella* before the treatment, but became *Bacteroides*, *Enterococcus* and *Veillonella* after the treatment, respectively.

**FIGURE 3:**
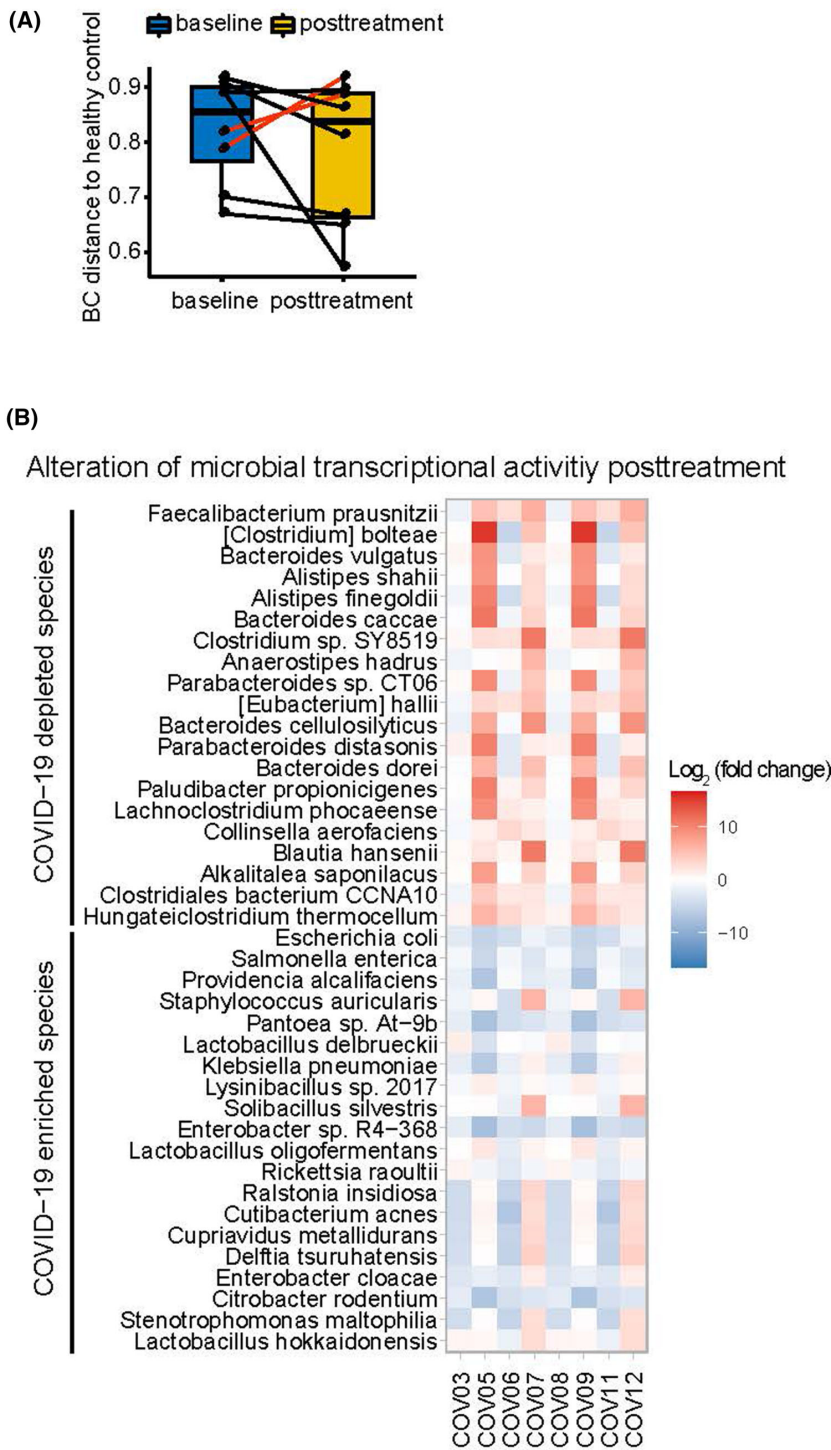
Shifts of the gut microbiome in COVID‐19 before and after the treatment. (A) Dissimilarity of the gut microbiota of COVID‐19 patients to that of healthy controls before and after treatment. The microbiota dissimilarity was calculated as Bray–Curtis dissimilarity. (B) The alteration of COVID‐19 enriched species (C) and depleted species of transcriptional activity after treatment

In conclusion, we identified substantial COVID‐19‐associated gut and upper airway microbiota shifts, which were characterized by great inter‐personal and inter‐timepoint variations. In addition to resolving the respiratory symptoms in COVID‐19 patients, a probiotics‐assisted therapy correlated with partial recovery of the microbiota perturbations (e.g., increased transcriptional activities of *Faecalibacterium prausnitzii* and *Roseburia hominis*, and decreased transcriptional activities of *Escherichia coli* and *Klebsiella pneumoniae*). These findings provided new insights into the gut and airway microbiome characteristics of COVID‐19 that may have significant clinical implications.

## Supporting information

Supporting information

Supporting information
