# Supplementary material for: The volatile and heterogeneous gut microbiota shifts of COVID‐19 patients over the course of a probiotics‐assisted therapy
Source: Clin Transl Med. 2021 Dec 28;11(12):e643. doi: 10.1002/ctm2.643 (PMC8713143; doi:10.1002/ctm2.643)
Supplement: Supplementary file 1 — Supporting information [file CTM2-11-e643-s001.docx]

**The volatile and heterogenous gut microbiota shifts of COVID-19 patients over the course of a probiotics-assisted therapy**

Chunyan Wu^1#^, Qian Xu^1#^, Zhan Cao^1#^, Dengdeng Pan^1^, Ying Zhu^1^, Sheng Wang^2^, Danping Liu^3^, Zhigang Song^3^, Wei Jiang^2^, Yumeng Ruan^4^, Yongkun Huang^5,6^, Nan Qin^1,4^*, Hongzhou Lu^3*^, and Huanlong Qin^1*^

^1^ Institute of Intestinal Diseases, Shanghai Tenth People’s Hospital, Tongji University School of Medicine, 301 Middle Yanchang Road, Shanghai 200072, China

^2^ Department of Critical Care Medicine, Shanghai Tenth People’s Hospital, Tongji University, 301 Middle Yanchang Road, Shanghai 200072, China

^3^ Department of Infectious Disease, Shanghai Public Health Clinical Center, 2901 Caolang Road, Shanghai 201508, China

^4^ Realbio Genomics Institute, Shanghai 200050, China

^5^ Department of Pediatrics, The First Affiliated Hospital of Kunming Medical University, Kunming, China

^6^ Yunnan Key Laboratory of Clinical Medicine, Kunming, China

#These authors contributed equally to this work.

*Correspondence should be addressed to Huanglong Qin (qinhuanlong@tongji.edu.cn), Hongzhou Lu ([luhongzhou@fudan.edu.cn](mailto:luhongzhou@fudan.edu.cn)) and Nan Qin ([qinnan001@126.com](mailto:qinnan001@126.com)).

**Materials and Methods**

**Recruitment of participants**

This study recruited 13 hospitalized COVID-19 patients, 15 hospitalized patients with community acquired pneumonia (pneumonia controls) and 15 healthy controls （previous study ^1^） (Table S1). SARS-CoV-2 infection was detected by dual RT-PCR test targeting two different regions of the RdRp gene. Non-COVID-19 pneumonia controls were patients admitted with community-acquired pneumonia who were tested negative for SARS-CoV-2. Patients with COVID-19 and pneumonia controls were admitted to the Shanghai Public Health Center (SHPHC, Shanghai, China). Healthy controls were individuals with no past medical history or history of antibiotic intake in the past 3 months recruited from the general population and tested negative for SARS-CoV-2. All subjects were recruited from February 18 through March 6, 2020. The inclusion criteria were the following: 1) 18 years of age or older; 2) comparable numbers of both genders; 3) patients must be able to sign informed consent and understand the possible risks of probiotic intervention; 4) willing to provide stool and blood samples on time; 5) respiratory distress, respiratory rate ≥30 beats/min, oxygen saturation level ≤ 93% in resting state and partial pressure of oxygen (PaO2)/oxygen concentration (FiO2) ≤ 300 mmHg (1 mmHg = 0.133 kPa). The exclusion criteria were the following: 1) dysphagia or gastrointestinal obstruction; 2) patients with autoimmune diseases, or with extremely low immunity, or with other diseases requiring systemic use of immunosuppressive drugs or hormone therapy; 3) human immunodeficiency virus (HIV) positive or active hepatitis b or c infection; pregnant or nursing women; individuals who were not suitable for study enrolment after clinical assessment (on the ground that the therapy might not benefit the patients or was too risky for the patients). Written informed consent was obtained from all participants or their families. All procedures were performed in compliance with the Declaration of Helsinki.

Epidemiological, clinical, radiological characteristics, laboratory, and treatment data were obtained from the electronic medical records. Clinical information included demographic data, medical history, exposure history, underlying comorbidities, symptoms, signs, laboratory findings, chest computed tomographic (CT) scans, and treatment measures (ie, antiviral therapy, antibiotics therapy, respiratory support). Fecal and nasopharyngeal swabs from patients with COVID-19 were collected weekly once the patients received a probiotics-assisted therapy comprising antiviral agents, antibiotics, and probiotics.

**Probiotics-assisted therapy**

All patients were admitted to the ICU and received antiviral therapy and other supportive care, while some patients received antibiotic treatment, antifungal treatment, glucocorticoid, and oxygen support at the appropriate situation. Patients were given 4 tablets of probiotics daily for two weeks. Each probiotics tablet contains at least 50 billion colony forming units of bacterial cells comprising the following strains: *Bifidobacterium lactis* subsp. HNO19, *Lactobacillus casei* subsp. Lc-11, *Lactobacillus plantarum* subsp. Lp-15, *Bifidobacterium lactis* subsp. B420, *Bifidobacterium longum* subsp. BL05, *Lactobacillus format* subsp. Lg-36, *Lactobacillus rhamnosus* subsp. Lr-32, *Lactobacillus paracasei* subsp. Lpc-37, and *Lactobacillus salivarius*.

**DNA and RNA extraction**

Fresh fecal and nasopharyngeal swabs were collected from all enrolled individuals, separated into two aliquotes (fresh-frozen, RNAlater), and then stored at -80℃ within two hours. DNA was extracted from samples using QIAamp Fast DNA Stool Mini Kit (Qiagen, Hilden, Germany) according to the manufacturer’s instructions. The concentration of DNA was measured by NanoDrop (Thermofisher, USA). Total RNA from samples was extracted using QIAamp Viral RNA Mini Kit (Qiagen, Hilden, Germany).

**16S rRNA gene sequencing analysis**

DNA from samples was amplified using primers targeting the V3-V4 regions of 16S rRNA gene (341F and 806R). Amplicon libraries were sequenced on Illumina Miseq platform for paired end reads of 300bp. Raw sequence reads were filtered and quality-trimmed as follows: (1) trimming low-quality base (average quality score <20); (2) removing reads shorter than 50 base pairs; (3) removing sequencing adapters; (4) remove reads with ambiguous N base >3. Paired-end reads were merged by pandaseq ^2^ with overlap. Considering the sequencing saturation and integrity of each sample, 42,000 merged sequences were randomly calculated for each sample. Merged sequences were clustered to generate operational taxonomic units (OTUs) clustering at the 97% similarity level after chimeras filtering, using UPARSE ^3^. A representative sequence of each OTU was assigned to a taxonomic level in the RDP database ^4^ using the RDP classifier at an 80% confidence level ^5^.

**Metatranscriptomic** **analysis**

No rRNA depletion steps were performed due to the limited biomass of the starting material. Double-stranded cDNA was synthesized with the QuantiTect Rev. Transcription Kit (Qiagen, Hilden, Germany) and NEBNext RNA Second Strand Synthesis Module Kit (New England BioLabs, Ipswich, Massachusetts, USA), and amplified with the Hieff NGS One Pot DNA Library Prep Kit for Illumina (Yeasen, Shanghai, China). The quality of all libraries was evaluated using an Agilent bioanalyser (Agilent Technologies, USA) with a DNA LabChip 1000 kit. Metatranscriptomic sequencing of samples was conducted on Illumina Hiseq 4000 platform with 150bp paired-end read length.

In total, we obtained 445 Gb of raw data (15.3 ± 6.7 Gb per sample). Raw reads of metatranscrptomic sequencing were processed to remove low-quality reads and adaptor contamination. Bases with a quality score below 30 were trimmed from the 3’ end of reads, and reads were removed if they were shorter than 70 bp or mapped to the human genome. Finally, we obtained 380 Gb of clean data (average 13.1 Gb per sample), and the average proportion of high-quality reads was approximately 97.14%. Clean reads were taxonomically profiled using Kraken ^6^ with default parameter settings. HUMAnN2 ^7^ was used to calculate the relative abundance of metabolic pathways in MetaCyc database. ShortBRED ^8^ was used to quantify the abundance of antibiotic resistance genes and virulence genes against the CARD database ^9^ and Virulence Factors Database ^10^. All analyses were performed on gene abundances normalized to reads per kilobase per million reads (RPKM). Strain-level profiling was performed with StrainPhlAn ^11^. For each sample, the clean reads were first mapped against the markers by Bowtie2 and then the consensus sequence was produced according to the mapping result. Finally, the extracted consensus sequences of references and samples were multiply aligned by MUSCLE ^12^, and the phylogenetic trees were built by RAxML ^13^ (parameters: -m GTRCAT and -p 1234).

**Statistics**

To detect significant differences in relative abundance of taxon features, the nonparametric Wilcoxon test (wilcox.test in R) was performed with false discovery rate (FDR) <0.05 (Benjamini-Hochberg), and the enrichment group was then determined according to the higher rank-sum. Spearman’s rank correlation was computed in R software (cor.test in R). P values were Bonferroni corrected for the number of tests performed in each case. All correlations mentioned were significant after correction for multiple testing (FDR < 0.05). Non-metric multi-dimensional scaling (NMDS) of Bray-Curtis similarities was performed within the vegan package of R software. In order to examine the overall community dispersion, we analyzed the taxa data using permutational analysis of multivariate dispersions (PERMDISP) with the R software package ‘PERMDISP2’. We also performed pairwise comparisons of community dispersion among participants in each group using PERMDISP2 with Bonferroni corrections to the overall per-estuary significance level of 0.05.

**References**

1. Gao, R.*, et al.* Gut microbiota dysbiosis signature is associated with the colorectal carcinogenesis sequence and improves the diagnosis of colorectal lesions. *Journal of gastroenterology and hepatology* **35**, 2109-2121 (2020).

2. Masella, A.P., Bartram, A.K., Truszkowski, J.M., Brown, D.G. & Neufeld, J.D. PANDAseq: paired-end assembler for illumina sequences. *BMC bioinformatics* **13**, 31 (2012).

3. Edgar, R.C. UPARSE: highly accurate OTU sequences from microbial amplicon reads. *Nature methods* **10**, 996-998 (2013).

4. Cole, J.R.*, et al.* Ribosomal Database Project: data and tools for high throughput rRNA analysis. *Nucleic acids research* **42**, D633-D642 (2014).

5. Kuczynski, J.*, et al.* Using QIIME to analyze 16S rRNA gene sequences from microbial communities. *Current protocols in microbiology* **27**, 1E. 5.1-1E. 5.20 (2012).

6. Wood, D.E. & Salzberg, S.L. Kraken: ultrafast metagenomic sequence classification using exact alignments. *Genome biology* **15**, 1-12 (2014).

7. Franzosa, E.A.*, et al.* Species-level functional profiling of metagenomes and metatranscriptomes. *Nature methods* **15**, 962-968 (2018).

8. Kaminski, J.*, et al.* High-specificity targeted functional profiling in microbial communities with ShortBRED. *PLoS computational biology* **11**, e1004557 (2015).

9. Jia, B.*, et al.* CARD 2017: expansion and model-centric curation of the comprehensive antibiotic resistance database. *Nucleic acids research*, gkw1004 (2016).

10. Chen, L.*, et al.* VFDB: a reference database for bacterial virulence factors. *Nucleic acids research* **33**, D325-D328 (2005).

11. Truong, D.T., Tett, A., Pasolli, E., Huttenhower, C. & Segata, N. Microbial strain-level population structure and genetic diversity from metagenomes. *Genome research* **27**, 626-638 (2017).

12. Edgar, R.C. MUSCLE: multiple sequence alignment with high accuracy and high throughput. *Nucleic acids research* **32**, 1792-1797 (2004).

13. Stamatakis, A. RAxML version 8: a tool for phylogenetic analysis and post-analysis of large phylogenies. *Bioinformatics* **30**, 1312-1313 (2014).


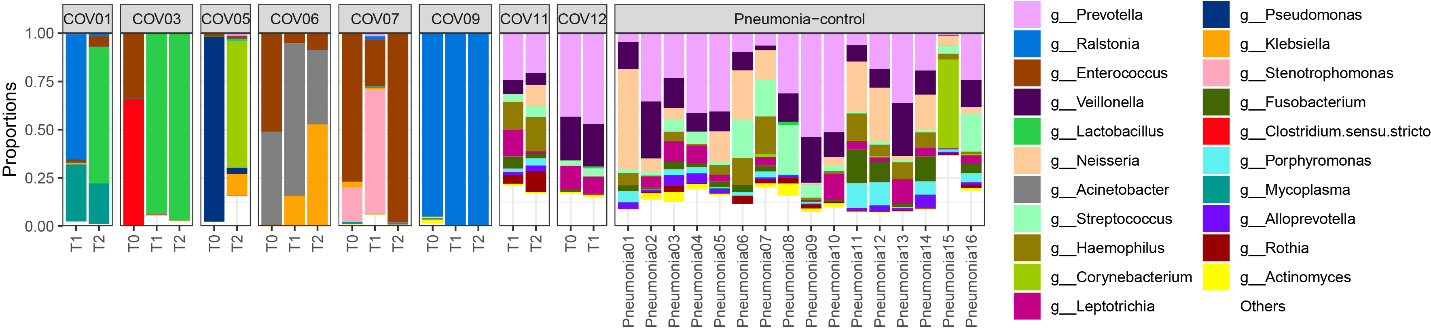


Figure S1. Relative abundance of top 20 taxonomic genus in COVID-19, and non-COVID-19 pneumonia controls in the upper airway.


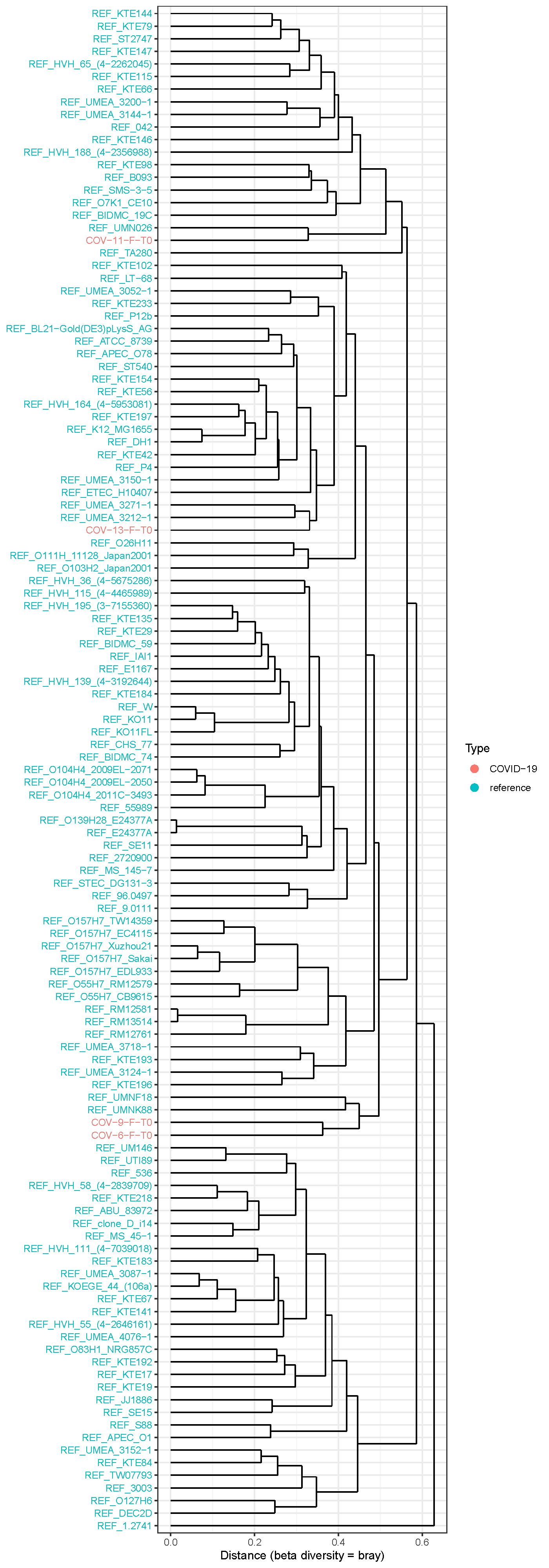


Figure S2. Strain-level cluster tree of *Escherichia coli* based the BC (Bray-Curtis) distance of presence/absence gene-family profile.


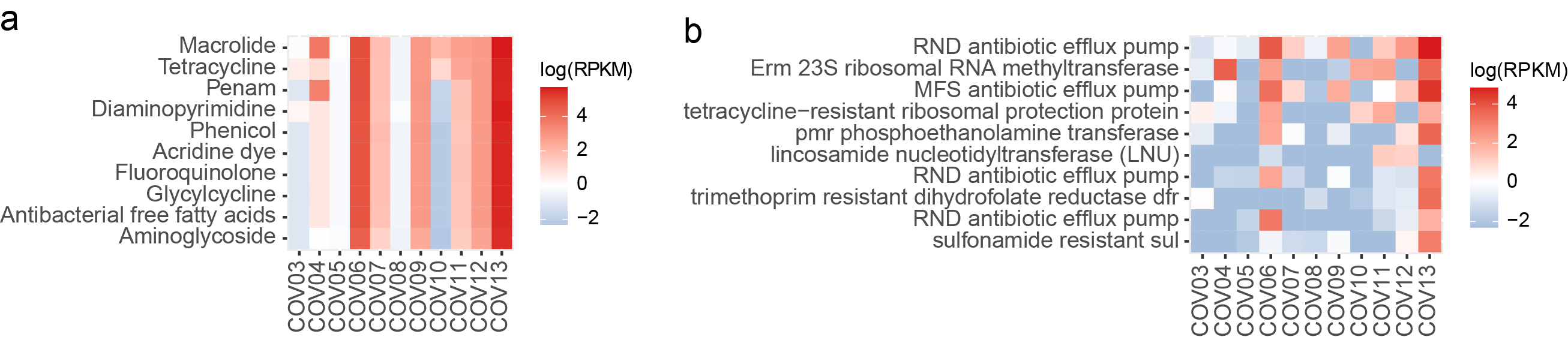


Figure S3. Heatmaps showing the top 10 most abundant antibiotic resistance targets (a) and antibiotic resistance genes (b).


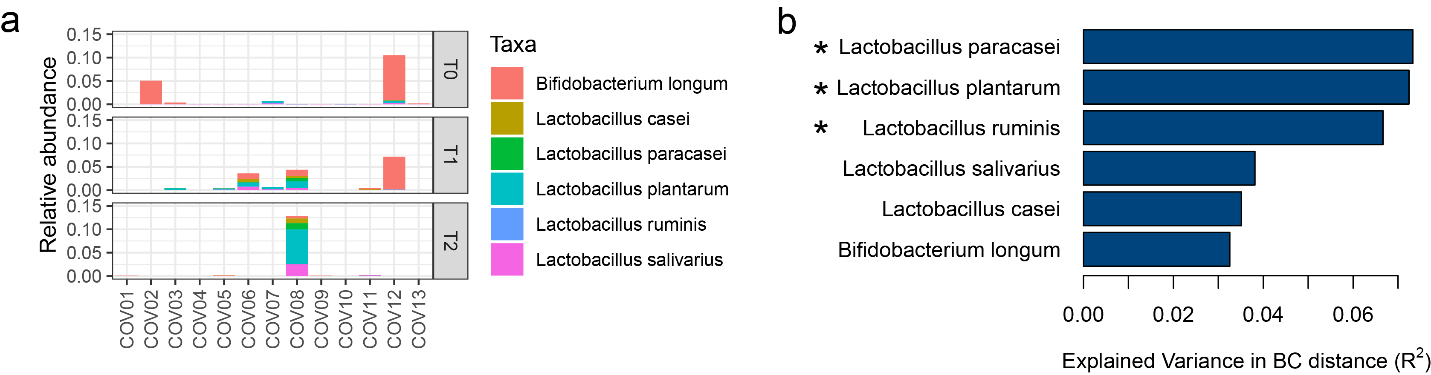


Figure S4. Influence of probiotics in gut microbiota of COVID-19 patients. (a) Bar plot showing the alteration of relative abundance of probiotics before and after treatment in COVID-19 patients. (b) Bar plot illustrating the probiotics found to be significantly associated with gut microbial variations. The variations were derived from between-sample Bray-Curtis distances. Size effect and statistical significance were calculated by PERMANOVA (Adonis). Significant associations were marked by * (FDR < 0.05).
